# The relationship between socioeconomic indicators during pregnancy and gynecological appointment at any time after childbirth

**DOI:** 10.1186/s12939-015-0191-x

**Published:** 2015-08-12

**Authors:** Alexandre Faisal-Cury, Julieta Quayle, Tatiana Marques, Paulo Rossi Menezes, Alicia Matijasevich

**Affiliations:** Department of Preventive Medicine, University of Sao Paulo School of Medicine, Av. Dr. Arnaldo 455, São Paulo, 01246-90 Brazil; Rua Dr Mário Ferraz 135/42, São Paulo, 01453-010 Brazil

**Keywords:** Inequality, Gynecological appointment, Maternal postnatal visits, Common mental disorders, Perinatal depression, Maternal health

## Abstract

**Background:**

The rates of receipt of postnatal care vary widely between high and low-middle income countries. This study aimed to examine the association between indicators of socioeconomic status during pregnancy and gynecological appointment at any time after childbirth (GA).

**Methods:**

a prospective cohort study with pregnant women recruited from 10 primary care clinics of the public sector in the city of São Paulo, Brazil. Socioeconomic characteristics and obstetric information were obtained through a questionnaire administered during pregnancy and in the postpartum period. Adjusted risk ratios (RR) with 95 % confidence intervals (CI) were calculated using Poisson regression.

**Results:**

Eight hundred and thirty one pregnant women were included in the study during the antenatal period and 701 were re-assessed during the postnatal period. Among them, 283 (59.6) attended a gynecological consultation. After adjusting for covariates, higher socioeconomic status during pregnancy was associated with greater risk of having a GA (RR:1.23, CI 95 %:1.05:1.45 for family per capita monthly income; RR:1.19, CI 95 % 1.01:1.40 for asset score).

**Conclusion:**

In this sample, the attendance for GA was above average and women with higher socio-economic status were more likely to have receipt of such care. Special efforts should be made to improve the attendance and frequency of gynecological consultations after childbirth among poorer women.

## Background

The postnatal period offers a unique opportunity to address the health care needs of women [1, 2]. The WHO guidelines recommend essential care for all mothers and their newborns beginning within 6 to 12 h after birth and follow-up visits from 3 to 6 days, at 6 weeks, and then at 6 months [3]. Postnatal care helps to identify complications, promote healthy behaviors, ensures the establishment of successful infant feeding, links the mother to family-planning services and the baby to child health care as well as fostering the development of good maternal-infant relationships [4]. However, the proportion of women who receive health care during this critical period varies widely between high and low income countries. Evidence indicates that health care coverage in the postpartum period is close to 90 % in developed countries [5], while rates are much lower in low- and middle-income countries. In general, the rate of attendance to postpartum visits is low varying from 34.6 %, in Congo [6] to 70 %, in Lebanon [7] and China [8]. Although studies have focused on the postpartum visit, usually limited at the first 3 or 4 months, a gynecological appointment at any time after childbirth (GA) is important for several reasons. Assessment of breastfeeding, safe contraception, urinary incontinence, and mental health monitoring/counseling are among the health care that can be offered for every woman after childbirth. The American College of Obstetricians and Gynecologists states that periodic assessments offer an excellent opportunity for obstetricians and gynecologists to provide preventive screening, evaluation, and counseling and recommends routine assessments in primary and preventive care for women based on age and risk factors [9].

Studies performed in several countries such as Brazil [10], Spain [11], Egypt [12], Russia [13] and England [14] have shown inequalities in maternal care either during pregnancy or after childbirth. Health seeking behavior for antenatal care is more frequent among educated, urban and higher socioeconomic status women [15] and women with higher socio economic status attend more postnatal appointments than women with lower socioeconomic status [16, 17]. In Brazil (a middle income country), data on postpartum maternal care visits is scarce. Two studies have shown quite large differences in coverage. In one study, from the State of Ceará, Northeast Brazil, only 2 % of women reported having received care up to 6 months after delivery [18], while in another study from the city of Pelotas, in Southern Brazil, 77 % of women received postpartum care before the third month after delivery [19] reflecting the continental differences present in health care in the country. Data on inequalities in gynecological assessment conducted months after childbirth are lacking.

Since 1989, The Brazilian Constitution guarantees to all citizens, a health care designed to be integral (offering care for all health problems), universal (covering anyone independent of contribution or employment status), and free (no user fees of any kind) through the Unified Health System (SUS). The public sector provides care for 74 % of the population [20]. Despite socio-economic progress made in recent decades, Brazil still has major contradictions. On one hand, according to 2008 data, over 90 % of the population receives the care requested [20] and in addition, several interventions implemented in primary health care are close to achieve universal coverage [21]. Healthcare coverage and utilization in Brazil appears to have become increasingly equitable over the past 10 years [22]. On the other hand, maternal mortality is still high [23]. Three-quarters of all maternal deaths occurred in the postpartum period [24]. Moreover, there are some concerns about the Brazilian health system’s ability to improve equity in healthcare access [25]. Racial and socioeconomic disparities are the main obstacles to equitable assistance in health.

This study aimed to examine the relationship between socioeconomic indicators during pregnancy and gynecological appointment at any time after childbirth, controlling for covariates. We hypothesized that women with lower socio-economic status are less likely to attend a GA.

## Methods

### Study design and sample

This study was a prospective cohort study that was conducted between May 2005 and January 2006 with eight hundred and thirty one pregnant women recruited from 10 primary care clinics of the public sector in three administrative districts in the Western area of the city of São Paulo, Brazil. The study area comprised a heterogeneous population of approximately 250,000 inhabitants where people with high, medium and low income live near each other. Public primary care clinics offer free antenatal care for all women living in their catchment areas. Prenatal care is offered regularly to low risk pregnant women, usually once a month, and generally starts as soon as the woman goes to the clinic for a pregnancy test. High risk pregnant women receive prenatal care in regional hospitals. There were two public hospitals in the study area, accounting for approximately 2,000 deliveries per year. Women also attended the primary care clinics after childbirth. Pregnant women between 20 and 30 weeks of pregnancy, whose conception occurred naturally, who were 16 years of age or older, who had singleton pregnancies, and who were receiving prenatal care in primary care clinics in the study area were considered eligible for this study. Pregnant women with a history of psychosis were excluded from the study. More details of the study sample have been described elsewhere [26]. Pregnant women were interviewed at primary care clinics. Postpartum women were interviewed at home after childbirth (mean time of interview: 11.1 months, SD: 2.3 months). Almost three-quarters of the women were interviewed between 6 and 12 months after childbirth, and 27.6 % were evaluated up to 14 months after childbirth.

### Main exposure variable

The two main exposure variables used for the assessment of pregnant women socio-economic indicators were “assets score” (in tertiles) and “monthly family income per capita” (0–59, 60–113 and 114–810 USD). Household assets measured included electricity, plumbing, computer, television, cable television, bathroom, telephone and refrigerator. An “asset-based score” (AS) was created using principal component analysis. The primary component was used to generate tertiles. Monthly family income per capita was defined as the monthly family income divided by the number of adults and child living in the house. Both were self-assessed through direct questions present in the questionnaire.

### Main outcome variable

Gynecological appointment at any time after childbirth (GA) was evaluated through a direct question during home interviews (“Did you a have any gynecological appointment at any time after childbirth?”). Time of gynecological appointments was also assessed (“How long after childbirth (in months) have you had a gynecological appointment?”). In the Brazilian public primary care clinics, gynecological assessment is performed by physicians and includes activities such as screening of cervical cancer, contraception advice, treatment of gynecological diseases.

### Questionnaire

Sociodemographic and socioeconomic characteristics as well as obstetric information were obtained through a structured detailed questionnaire which was applied during the antenatal assessment. The information obtained included age, years of schooling, marital status, and skin color. Previous and current obstetric data included planning of last pregnancy, number of previous abortions, number of previous pregnancies, gestational age, birth weight and Apgar score at 5 min of life. A dichotomous measure (“yes-no”) classification of obstetric complications was developed. “Yes” was defined by the presence of a gestational age less than 37 weeks at birth, a newborn weight under 2500 g, or a 5 min Apgar score less than 7. In the postpartum period, breastfeeding was evaluated and defined as feeding the baby with breast milk, regardless of supplementing with other food. Breastfeeding length was ascertained through a single question to the mother: “How long have you breastfed?”. A dichotomous measure (“yes-no”) for the occurrence of breastfeeding up to 4 months was created. The presence of antenatal and postnatal depression was assessed with the Self-Report Questionnaire (SRQ-20), which was developed for screening common mental disorders in patients treated in primary care settings [27]. The SRQ-20 was validated in primary care centers in Brazil, with 85 % sensitivity and 80 % specificity [28]. The SRQ-20 has good psychometric properties for diagnosing antenatal and postnatal depression, performing even better than others instruments specifically designed for this purpose [29]. The optimal cut-off point for the SRQ-20 was set at 7/8 for the present study. Four groups were defined according to the presence of common mental disorders (CMD) during pregnancy and/or postpartum: group 1: absence of both antenatal and postpartum CMD; group 2: presence of antenatal CMD only; group 3: presence of postpartum CMD only; group 4: presence of both antenatal and postpartum CMD.

### Procedures

During the study period, trained research assistants went to the primary care clinics and approached pregnant women who came for antenatal care. Eligible women were invited to participate. Those who agreed and signed an informed consent form for participation were then interviewed. The same group of research assistants administered the questionnaire and the SRQ-20 in the postnatal period through home interviews. There were no financial incentives for participation either during pregnancy or after childbirth. The participants answered these instruments up to 14 months after childbirth. The Ethics Committee of the University of São Paulo, School of Medicine approved the research project.

### Statistical analysis

Descriptive frequencies were summarized, and all variables studied were categorized. Crude and adjusted risk ratios (RR), with 95 % confidence intervals (95 % CI) were calculated using Poisson regression with robust variance to examine the associations between each socioeconomic variable with GA. Adjusted analyses were used to examine the association between the two main exposure variables (assets score and monthly family income per capita during pregnancy) and GA controlling for potential confounding variables. We examined the effect of each exposure variable on postnatal gynecological assessment accounting for potential confounders by using four different models: [1] model 1: crude association; [2] model 2: model 1 plus adjusting for demographic variables (mother’s age, marriage status, years of education, skin color and time of assessment after childbirth); [3] model 3: model 2 plus adjusting for obstetrical characteristics (number of pregnancies, previous miscarriage, pregnancy planning, obstetrical complications, cesarean section, episiotomy, 4 months of breastfeeding, perinatal depression), and [4] model 4: model 3 plus adjusting for the other socio-economic variable (monthly familiar income or asset score). Covariates were identified a priori based on previous research on GA and socioeconomic factors. To be included as potential confounders variables had to be associated with socioeconomic factors and GA with a P level <0.2. Statistical associations were assessed with likelihood ratio tests. Statistical analyses were performed using STATA 11 software.

## Results

Eight hundred and sixty-eight eligible pregnant women were identified, and 831 (95.7 %) of these women were included in the study during the antenatal care period. Of these women, 701 (84.4 %) were re-assessed during the postnatal period. The average age of these women was 25 years, and 147 women (21 %) were less than 20 years of age. Almost three-quarters (73.3 %) of the women were living with a partner. Just over half (53 %) of them were white, and a similar proportion had more than eight years of education. The average family monthly income was 440 U.S. dollars. One hundred and ninety-six participants (28.0 %) presented antenatal CMD as evaluated by SQR-20 (Table 2). Among the 701 postpartum women, 418 (59.6 %) had a GA. Thirty seven eligible pregnant women refused to participate for several reasons (lack of time, would not return for prenatal appointments). In comparison with postpartum women, pregnant women who did not return after delivery had similar family income, were less educated and had more common mental disorders.

In the bivariate analysis, family per capita monthly income, assets score, years of schooling, skin color and time of interview were associated with having a GA (Table 1). In contrast, none of the delivery and postpartum data was associated with GA (Table 2).

**Table 1:** Socio-demographic, socioeconomic, and other health-related characteristics of the sample, according to the presence of gynecological appointment any time after childbirth (GA)

		GA	
	N	N (%)	P level
Family per capita monthly income (USD)			0.004
0 – 59	224	115 (51.3)	
60 – 113	232	138 (59.5)	
114 -810	238	160 (67.2)	
Asset score (tertiles)			0.005
First	219	119 (54.3)	
Second	227	158 (57.0)	
Third	205	141 (68.8)	
Skin colour			0.009
White	327	212 (64.9)	
Black/mixed/other	374	206 (55.1)	
Marriage Status			0.54
Unmarried	187	115 (61.5)	
Married	514	303 (58.9)	
Mother’s age			0.86
16-19	147	89 (60.5)	
20-29	388	233 (60.0)	
30-44	166	96 (57.8)	
Time of interview (in months after childbirth)			0.048
6-9	99	61 (61.6)	
10-12	408	256 (62.7)	
13-16	193	101 (52.3)	
Previous miscarriage			0.07
No	537	330 (61.4)	
Yes	164	88 (53.6)	
Number of pregnancies			0.14
1	246	154 (62.6)	
2	215	133 (61.8)	
3 or more	240	131 (54.6)	
Years of education			0.018
0-8	326	179 (54.9)	
9 or more	375	239 (63.3)

**Table 2:** Delivery and postpartum data of the sample, according to the presence of gynecological appointment any time after childbirth (GA)

		GA	
	N	N (%)	P level
Obstetric complication			0.29
No	568	344 (60.5)	
Yes	133	74 (55.6)	
Low birth weight			0.64
No	648	388 (59.9)	
Yes	53	30 (56.6)	
Preterm			0.25
No	571	347 (60.8)	
Yes	118	65 (55.1)	
Cesarean Delivery			0.13
No	483	279 (57.7)	
Yes	218	139 (63.7)	
Forceps			0.28
No	584	343 (58.7)	
Yes	117	75 (64.1)	
Episiotomy			0.45
No	408	238 (58.3)	
Yes	291	178 (61.7)	
Breastfeeding (4 months)			0.64
No	480	129 (58.3)	
Yes	221	189 (60.2)	
Perinatal Depression			0.75
No	395	239 (60.5)	
Antenatal only	110	68 (61.8)	
Postnatal only	87	48 (55.1)	
Antenatal and postnatal	109	63 (57.8)	

In the multivariable analysis, after adjusting for covariates, higher socio-economic status women have approximately 20 % greater chance of having a GA in comparison with women with low socio-economic status (Table 3).

**Table 3:** Crude and adjusted associations of socioeconomic variables with the presence of gynecological appointment any time after delivery (GA)

	Model 1: unadjusted	Model 2: Model 1 plus mother’s characteristics	Model 3: Model 2 plus Obstetric Complications	Model 4: Model 3 plus SE variable
	RR	RR/CI 95 %	RR/CI 95 %	RR/CI 95 %
Family per capita monthly income (USD)	p = 0.004	p = 0.005	p = 0.007	p = 0.008
0 – 59	1.0	1.0	1.0	1.0
60 – 113	1.15 (0.98:1.37)	1.15 (0.98:1.36)	1.15 (0.97:1.35)	1.12 (0.95:1.32)
114 -810	1.31 (1.12:1.53)	1.29 (1.10:1.51)	1.29 (1.11:1.51)	1.23 (1.05:1.45)
Asset Score (tertiles)	p = 0.003	p = 0.005	p = 0.007	p = 0.009
First	1.0	1.0	1.0	1.0
Second	1.05 (0.89:1.23)	1.02 (0.87:1.19)	1.03 (0.88:1.21)	1.01 (0.86:1.19)
Third	1.26 (1.08:1.47)	1.20 (1.02: 1.40)	1.25 (1.08:146)	1.19 (1.01:1.40)

Family per capita monthly income

Model 1: crude association

Model 2: adjusted by model 1 plus demographic characteristics (skin color*, marriage status, mother’s age, years of education, time of interview after childbirth)

Model 3: adjusted by model 2 plus obstetric characteristics (cesarean delivery*, previous miscarriage*, perinatal depression, number of pregnancies, planning of pregnancy, obstetric complication, , episiotomy, breastfeeding up to 4 months)

Model 4: adjusted by model 3 plus asset score

Asset score

Model 1: crude association

Model 2: adjusted by model 1 plus marriage demographic characteristics (skin color*, years of education*, marriage status, mother’s age, time of interview after childbirth)

Model 3: adjusted by model 2 plus obstetric characteristics (obstetric complication*, cesarean delivery*, previous miscarriage*, perinatal depression, number of pregnancies, planning of pregnancy, episiotomy, breastfeeding up to 4 months)

Model 4: adjusted by model 3 plus family monthly percapita income

*:remained in the model

## Discussion

Our study assesses gynecological appointment after childbirth in a sample of pregnant women from a public healthcare sector in a middle-income country. The main results show that attendance for GA is above average (59.6 %) and lower socio-economic status women are less likely to have any gynecological assessment after childbirth. As far as we know this is the first study in Brazil about gynecological assessment up to 14 months after childbirth and it confirms that inequalities is an independent risk factor for GA.

Low rates of postnatal visits are quite common in low-income countries. In Brazil, there is a paucity of studies in this subject. The rate of maternal postnatal visits varies between 2 % [18], in Ceará state, located in the Northeast to 77 % of postpartum women [19], in Pelotas city, in the Southern of Brazil. The discrepancy may be attributable to economic difference between regions. Ceará is one of the nine states in the Northeastern of Brazil, the poorest region of the country. In contrast, the city of Pelotas is economically developed. In our study we have found a rate of almost 60 % of GA. In comparison with the Pelotas study, our lower rate of attendance during the months following childbirth may be attributable to the specific characteristics of our sample, comprised of many adolescents, black and single mothers, which might have contributed to our results, even considering that São Paulo is economically strong and offers good health coverage to the population. On the other hand, the sample of postpartum women in Ceará study was poorer with 56 % of them being illiterate. Moreover, 78 % of these postpartum women had a family income of less than 1 minimum salary per month (less than 300 dollars in current exchange rate; that is now 1 dollar = 3 reais).

Regarding to the studies performed in other countries, there is a large variation of rates of maternal postnatal visits. The followings rates have been mentioned: 35 %, for Congo [6], 68.6 %, for China [8], and 85 %, for Unites States of America [2]. Comparison of rates of attendance to maternal postnatal visits among countries is difficult because the timing (as well content) of maternal postnatal visits varies widely. The span of time for postpartum consultation used in several studies varies between 48 h [30, 31], 6 weeks [6] and 6 months [2] after childbirth. Nevertheless, studies agree that women living in disadvantaged socio-economic conditions [32] are at high risk of not receiving postnatal care. It is generally recognized that insufficient financial means may act as a barrier to the utilization of health care services [33]. Socially deprived postpartum women may have difficulties in seeking medical help after childbirth due to the costs of transportation to the health center. Moreover health facilities may be located far away from home and may not have available care on weekends for employed women. As we mentioned before there is a lack of studies addressing the frequency and determinants of gynecological evaluation long time after childbirth. But it seems that the same pattern observed during the antenatal and postnatal care persists, being lower socio-economic status women less likely to have gynecological assistance.

This explanation must be contextualized within the structure of Brazil’s public health care system. The Brazilian Unified Health System ensures free access to all types of health care for the entire population and no fee is charged at any point of the process for attending antenatal or postnatal governmental health services, as well as hospital facilities for delivery. Therefore, the lower frequency of GA among poorer women may be related not only to economic barriers, but also to cultural or educational factors. Postpartum women may consider the care after childbirth less important than prenatal care since their main goal could be the birth of a healthy baby and not self-care. They may even consider the quality and content of gynecological care inadequate or insufficient. As a matter of fact, there is evidence that the utilization of healthcare services during the postnatal period is influenced by a number of factors including socio-cultural beliefs of women and their families regarding the importance of postnatal care for the mother [34].

There are a few implications of our results. It could help health policy planners to decrease inequalities in women’s health indicators as well to promote equitable access to gynecological services [35]. Nevertheless, the best cost-effectiveness strategies to reach these goals need to be confirmed through randomized trials. There are already evidences in favor of such approach during pregnancy, childbirth and in the 28 days following birth in low and middle income countries [36]. For example, interventions such as “Familias Sanas” [37] have the potential to increase rates of postnatal attendance among low income mothers. In this randomized control trial, prenatal partners helped pregnant Latinas in the U.S. to navigate the health care system, advocate for themselves, and understand the importance of the postpartum visit. Women in the intervention group were about 2.5 times more likely to attend postpartum visits compared with those in the control group. Notably, since prenatal partners used in this study were social work students, there is the possibility that the intervention can be replicated by training the staff (e.g. nursing assistants) in existing public health clinics in the Brazilian health system.

### Methodological considerations

The strengths of our study include a prospective evaluation of low-income pregnant women attending antenatal care in Primary Care Units in the city of São Paulo, a large urban center in a middle-income country up to 14 months after childbirth. Second, regarding our exposure variables, we have assessed not only familial income during pregnancy but also a wealth score, an index based on the ownership of household assets. Direct data on income are subject to reporting bias. Therefore we have used asset score as one indicator of women’s socio-economic status. Family income in Brazil is mainly defined by partners’ wage salary, and it is a more transient indicator of socioeconomic status, while wealth scores reflect a more stable pattern of consumption. Finally, data about perinatal CMD was included in the analysis. Perinatal CMD is probably a confounding considering that lower income pregnant women are at higher risk of being depressed [38] and depressed postpartum women are less likely to seek for health care [39].

Some limitations of this study deserve attention. First, recall or reporting bias may occur given the number of months between delivery and the date in which women responded to the postpartum questionnaire. Our main outcome (recall of having a gynecological appointment any time after childbirth) was based on a single question. It would very be difficult to collect reliable data from different facilities. Frequently this is impossible considering the fact that the health system does not record information after birth on patients in a database. Although women may felt more comfortable over-reporting about having a medical appointment after childbirth, the frequency of reported GA is still low. Second, our study did not capture information on other factors that might have influenced these women’s gynecological attendance, such as women's knowledge on the importance of gynecological assessment, perceptions about the quality of health care available, and perceived barriers to the utilization of health care services. Third, our study included only users of primary health care clinics that traditionally in Brazil cover the poorest stratum of the population, including for antenatal care. This aspect may have sub-estimated the actual association between socio-economic status and attendance to puerperal consultation in Sâo Paulo. Nevertheless, the socio-demographic characteristics of our sample (age, race, education and number of children) are very similar to the Brazilian women who gave birth in 2005 [40]. Finally, our results cannot be generalized to the rest of the country (i.e. Brazil) or other low-middle income countries taking into account particularities in postnatal care systems.

## Conclusion

In this sample, the attendance for GA was 59.6 % and women with lower socio-economic status were less likely to have receipt of such care. Pregnant women with higher socioeconomic status presented an increased risk of approximately 20 % of having a GA in comparison with pregnant women with a lower socioeconomic status. The postpartum visit serves as a window of opportunity to provide further health care and education to women [2] as an opportunity for counseling about the importance of self-care in other periods of life than pregnancy. Although there is no clear consensus about the content of a scheduled contact with women after delivery, a late postnatal contact should be organized to link with ongoing care as currently provided for all women [41]. Despite the availability of near universal coverage for medical visits after childbirth in Brazil, as well in other countries, special efforts must be made to improve the attendance by poorer women to ensure access to essential services such as contraception, breast feeding education, and infant care. Moreover, there is a need for regular monitoring of gynecological assessment after childbirth and quality of care taking into account that there are inequities between different social groups. Possible barriers to medical care after birth (the cost of transportation, the distances from health facilities, the unavailabitility of care and women's beliefs and attitudes towards postpartum care) should be addressed. Specific measures such as increasing awareness and access to services through community-based programs especially for the rural, poor, and less educated mothers may increase postnatal attendance.
